# The process flow and structure of an integrated stroke strategy

**DOI:** 10.5334/ijic.888

**Published:** 2013-06-27

**Authors:** Emma F van Bussel, Thomas Jeerakathil, Augustinus J.P Schrijvers

**Affiliations:** BovenIJ Hospital, Amsterdam, The Netherlands; University of Alberta, Canada; Chair, Evaluation and Quality Improvement Pillar, Alberta Provincial Stroke Strategy, Alberta, Canada; Julius Center University Medical Center Utrecht, The Netherlands

**Keywords:** stroke, neurology, telestroke, telemedicine, integrated care, Canada

## Abstract

**Introduction:**

In the Canadian province of Alberta access and quality of stroke care were suboptimal, especially in remote areas. The government introduced the Alberta Provincial Stroke Strategy (APSS) in 2005, an integrated strategy to improve access to stroke care, quality and efficiency which utilizes telehealth.

**Research question:**

What is the process flow and the structure of the care pathways of the APSS?

**Methodology:**

Information for this article was obtained using documentation, archival APSS records, interviews with experts, direct observation and participant observation.

**Results:**

The process flow is described. The APSS integrated evidence-based practice, multidisciplinary communication, and telestroke services. It includes regular quality evaluation and improvement.

**Conclusion:**

Access, efficiency and quality of care improved since the start of the APSS across many domains, through improvement of expertise and equipment in small hospitals, accessible consultation of stroke specialists using telestroke, enhancing preventive care, enhancing multidisciplinary collaboration, introducing uniform best practice protocols and bypass-protocols for the emergency medical services.

**Discussion:**

The APSS overcame substantial obstacles to decrease discrepancies and to deliver integrated higher quality care. Telestroke has proven itself to be safe and feasible. The APSS works efficiently, which is in line to other projects worldwide, and is, based on limited results, cost effective. Further research on cost-effectiveness is necessary.

## Introduction

In the United States of America stroke is the third leading cause of death [1], and it is also a leading cause of functional impairment with 15–30% of survivors being permanently disabled [2]. The American Heart Association estimated that 780,000 strokes occur each year and annual costs are estimated to be 34 billion US dollar in the USA [1]. Stroke incidence increases with age. An aging population and unhealthy lifestyle choices threaten to cause a higher incidence of stroke and other chronic diseases in the years to come in the developed world [1, 3–5]. Risk factors are well known and guidelines for treatment and prevention are available [2, 6, 7]. Ischemic strokes account for approximately 87% of all strokes and hemorrhagic strokes for 10% [1].

A number of strategies are available to reduce the burden of stroke. Treatment of acute ischemic stroke with tPA (tissue plasminogen activator) increases neurological improvement. In eligible patients, the thrombolytic agent tPA has to be administered within 4.5 hours after onset of symptoms, the benefit is greater with earlier treatment [8, 9]. Of equal importance, timely secondary prevention is effective in reducing recurrent stroke in patients with stroke or TIA. Preventive measurements, such as blood pressure reduction, statin therapy and screening for additional risk factors, are proven effective [6].

Furthermore, coordinated stroke care results in improved outcomes and decreased length of hospital admission, as well as decreased costs. The implementation of telestroke as a component in a coordinated system of care increases the quality of care and the use of intravenous tPA at community hospitals [7]. Using the ‘National Institutes of Health stroke scale’, a reliable impression on the patients’ state via videoconferencing can be derived [10–12]. Patients and healthcare providers report high levels of satisfaction with the telestroke intervention. International literature shows that the number of rural TIA and stroke patients that receive tPA treatment increases through the use of telestroke [12, 13].

The acute and preventive care for stroke patients is complex and requires the efforts and skills of all members of a multidisciplinary team [7]. In low population density countries like Canada delivery of standardized stroke care is even more challenging.

Systemic deficiencies of stroke care together with the increasing cardiovascular risk factor burden in the aging population led to the creation of a national initiative to improve stroke care in Canada. In 2005 the Canadian Stroke Strategy (CSS) was created. Following the CSS the province of Alberta developed the Alberta Provincial Stroke Strategy (APSS).

The APSS is a multidisciplinary and comprehensive approach to improve stroke care across the province throughout the continuum. Its goals are to improve access to specialized stroke care and improve the quality of acute care and follow up. Telestroke is an important component of the strategy.

This article relates the experience of a single province within Canada. The central question is: What is the process flow and the structure of the care pathway of the Alberta Provincial Stroke Strategy? The components the APSS introduced to achieve the present pathway, including telestroke, are explained.

## Problem statement

Before the reformation of stroke care started in 2005, across Alberta, there was variable access to stroke care and little standardization of the approach to patients with a TIA. There was a gap between knowledge of best practice and actual practice for acute and non-acute stroke care, especially in rural hospitals. The situation before the creation of the APSS is reviewed here, to illustrate why a comprehensive intervention was adapted, and what elements were in the scope of the strategy.

In 2005 there were only four hospitals with neurologists, and only five hospitals that provided thrombolytic drugs to acute stroke patients, serving a population of 3.8 million Albertans, plus inhabitants of northern British Columbia (an adjoining province) and two northern Canadian territories which refer patients to Alberta. The province alone covers a 660,000 square kilometre area [14]. Other small hospitals in the province did not have neurologists, brain imaging possibilities or thrombolytic possibilities and rural physicians often lack training in neurological disorders. Often there was no timely communication with stroke neurologists.

People in northern Alberta and the adjoining territories had to travel up to 1500 kilometres to the capital city Edmonton to see a neurologist. In acute stroke situations their chance for timely thrombolysis was low only because of the travel time to the nearest hospital with the right expertise and equipment.

Besides, in many areas emergency medical services (EMS), lacked appropriate training in recognizing acute stroke symptoms and transported patients to the nearest hospital, irrespective of that facility’s stroke expertise. This led both to patients not being in time for tPA treatment, and to many interhospital transports.

Furthermore, there was a deficiency of timely cross disciplinary communication in regards to stroke care. In a broader sense there was no multidisciplinary management of stroke at the provincial level to ensure standards were being met and that access to appropriate care was available across the continuum.

There were no clear standards for the involvement of rehabilitation teams in the management of stroke inpatients, and transition of patients from inpatient or tertiary rehabilitation care to community living was not well developed.

The organization of preventive stroke care in Alberta initially was lacking. In 2005 there were three dedicated stroke prevention clinics providing information and care in the prevention of stroke, in the whole province.

## Methods

This article describes the care pathway of the APSS and its structure using the case study methodology by Yin [15]. A care pathway can be evaluated by structure quality, process quality and outcome quality. The structure quality of the APSS is evaluated, using relevant subjects found in the literature [16].

Data collection is based on Yin’s case study methodology. Yin [15] describes a case study as an empirical enquiry that investigates a contemporary phenomenon in depth and within its real-life context, especially when the boundaries between phenomenon and context are not clearly evident. The case is the Alberta Provincial Stroke Strategy and its telestroke program and the context is stroke care within Alberta. A characteristic of the case study is that it relies on multiple sources of evidence, with data to converge in a triangulation fashion. Yin distinguishes six sources of evidence: 1) documentation, 2) archival records, 3) interviews, 4) direct observations, 5) participant observations, and 6) physical artifacts or objects. In this case study physical artifacts were not used to support the findings. The five sources were used as follows.

*Documentation*: National and provincial policy papers [17, 18], local telestroke information [19, 20] protocols by the APSS [21–25] and an evaluation report published by the APSS [26] were collected. Reviews regarding telemedicine in stroke services worldwide were collected [12, 13].

*Archival records:* Organizational charts and a blueprint from the start of the APSS [27, 28] were used, as were statistics of the local and international population, its diseases and behavior [3–5, 14, 29]. International guidelines were used for evidence and evaluation of actions of the APSS [1, 2, 6–10].

*Direct observations*: The first author shadowed the stroke and telestroke service at the University of Alberta Hospital and the Walter C. Mackenzie Centre in Edmonton, during two weeks. She was involved with daily practice and had many meetings with professionals, managers and patients.

*Interviews*: During the two weeks at the Comprehensive Stroke Centre the first author had extensive interviews with stroke specialists and paramedic personnel working with telestroke. For a broader view on the Albertan health system she interviewed twelve key players of health organisations in Alberta; Alberta Health Services and its subdivisions and Alberta Health and Wellness.

*Participant observation*: One of the three authors (TJ) works in the telestroke services of Edmonton and also serves as Chair of the Evaluation Pillar of the APSS. He brought to the research team his experience.

Triangulation of the data was done by the first two authors. Linkage and synthesis of collected data were accomplished. There was an extensive process of data cleaning and troubleshooting resulting in a final analysis with homogenous information. Where possible, data trends were validated through comparison to existing datasets, such as those of the Canadian Institute for Health Information.

## Results

First the process flow of the integrated care pathway is described and outlined in flowcharts, with extra attention to telestroke. Then, the structure of the pathway of the APSS is described.

### Care pathway of the APSS

The APSS adopted a broad approach to address all elements of stroke care, including health promotion, disease prevention, acute care, rehabilitation and community reintegration. Box 1 presents a case to illustrate the process flow of stroke care in Alberta.

Flowcharts 1 and 2 give the general process flow of current stroke care, starting in the community, throughout the health system. The numbered hexagons indicate what factors were addressed and introduced by the APSS, and refer to the corresponding table (Table 1) explaining the improvements.

### Characteristics of the care pathway

#### Organizational commitment

The Canadian Stroke Network and the Heart and Stroke foundation of Canada launched in 2005 the Canadian Stroke Strategy (CSS) to improve stroke care in Canada. Strategies were developed provincially to make best use of resources and customize local priorities. The vision and targets of the stroke strategy of Alberta, align with the visions and targets of the health department of the provincial government (Alberta Health and Wellness (AHW)) and Alberta Health Services (AHS) [18, 26]. Because of the aligning visions and the national movement to improve stroke care, these organisations have given the APSS financial and operational support. Telehealth is supported through AHW and AHS, and additionally through donor contributions, Health Canada First Nations and Inuit Health Branch, and Canada Health Infoway strategic investments.

#### Pathway project management and accountability

The APSS has a clearly defined organizational structure and chain of accountability [27]. The Alberta Stroke Council (ASC), formed to oversee the activities of the APSS is a collaboration of stakeholders in stroke care in Alberta: AHW, AHS, the Heart & Stroke Foundation of Alberta North West Territories & Nunavut and clinical experts [26]. The ASC reports to AHW and maintains accountability for all deliverables. Four pillars (1. health promotion and disease prevention, 2. emergency services and acute care, 3. rehabilitation and community reintegration, 4. evaluation and quality improvement) and other committees report to the ASC. The Pillars 1–3 manage the pathway and strongly collaborate to integrate the different aspects of the pathway. The ASC contained executive level membership from regional health authorities and later from AHS which allowed it to make binding decisions on allocation of funds. As a result the ASC could be quite flexible and responsive to changing needs within the province and it could maintain its provincial perspective.

#### Format document

The APSS Blueprint is the initial overview document in which provincial capacities, gaps and proposed action plans are brought together. It provides the basis for the core business functions of the APSS and gives directions for moving forward to strengthen stroke services [28].

#### Professional content of pathway

At a national level Canadian best practice recommendations for stroke care [30] were developed and updated on a regular basis. These recommendations describe optimal care for stroke patients and best-practices for stroke prevention, medical care and recovery [17]. Most protocols of the APSS are based on these recommendations.

#### Multidisciplinary involvement

Stroke specialists in tertiary stroke centres initiated the APSS, and worked together with all stakeholders in stroke care in Alberta to make the strategy a success. They are all involved in the program and collaborate; care points are linked to one another in sequence resulting in a smoothened care process. This is evident in inpatient services between rehabilitation and medical teams, between primary care physicians and neurologists for TIA and acute stroke patients, and between administrators and all levels of health professionals. Furthermore, provincial protocols were created by multidisciplinary teams.

#### Variance management

Professionals working with stroke patients are trained and educated and use their experience to be at variance from the process flow if needed. Each patient is evaluated several times by different specialist of the multidisciplinary team and decisions are based on the combination of protocols and the experts experience.

#### Patient involvement

Representatives of stroke survivors (the Stroke Recovery Association of Alberta) were involved in the original creation of the provincial stroke strategy and hold membership on Pillar 3 of the strategy. From the first medical contact, patients are provided with disease information and the components of the care path. During follow up in the stroke prevention clinic and rehabilitation patients get tools and information to better lifestyle and set goals.

Telephone surveys of stroke survivors were conducted to evaluate and improve care. Quality of life, patient’s experience while in hospital and on return to community were measured.

#### Implementation of pathway

In 2005 Alberta was successful in creating the APSS. Stroke advocates, consisting of the Heart and Stroke Foundation, stroke physicians, and other stroke clinicians submitted a business case to the government of Alberta for a provincial stroke strategy. After the government of Alberta committed $20 million CAD over two years in 2005 the APSS came into being.

One of the first actions of the APSS was to create the Pillar structure that would form the foundation for practice standards and for implementation of services (see project management). In addition, the nine health regions formed stroke steering committees to manage the APSS funds and to guide the implementation of stroke services in the health regions in the areas of prevention, emergency and acute care services, rehabilitation and community reintegration.

There remained challenges in the implementation of the strategy. At the present time there is one health care delivery organisation—AHS—that works with the Government of Alberta. This organization deals with current and future developments of the APSS. In 2005 AHS did not exist, and the Alberta Stroke Council had to collaborate with the 9 health regions that preceded AHS to make the strategy a success. At the start not all regions identified improvements in stroke care as a priority in the context of many other obligations. These regions needed extra effort to join APSS to improve stroke services at the provincial level.

Furthermore, not all practitioners conformed to best practise standards in stroke care and it was challenging to expose many to these standards. In many areas the problem was not willingness but rather the inability to hire, retain and maintain the skills of administrators and health care practitioners particularly in remote areas. The engagement of primary care physicians and generalist physicians in the process was also a challenge in some areas of the province. This was particularly so in the acute telestroke encounter which produced a new kind of interaction between emergency physicians, emergency room staff, and stroke neurologists.

The APSS includes one of Alberta’s biggest telehealth initiatives: Telestroke. For its telestroke program, the APSS makes use of Alberta’s pre-existing telehealth network.

#### Maintenance and development of the pathway

The Blueprint is not static but changes over time as events and new ideas evolve [28]. The evaluation pillar of APSS developed a de-identified data repository containing multiple datasets. The database uses administrative data and a variety of new datasets to track all stroke related hospital contacts, and admissions, outcome and costs. The repository is the basis for regular evaluation and improvements. In an interim evaluation report [26] the APSS stated goals for the next period, based on outcomes of the evaluation in terms of quality, new identified needs and disease outcomes.

#### Quality management

To control the quality of stroke care and allow for multi-stakeholder review the Alberta Stroke Council was introduced. Furthermore, APSS Pillar 4—Evaluation and Quality Improvement—was charged with both evaluating the effectiveness of the strategy and with creating a quality management framework. The APSS governance recognized that to sustain the achieved gains there had to be continuing evaluation and measurement. The APSS identified fifty performance measures to evaluate progress made in delivery of stroke care [26]. For the near future the APSS still has goals to achieve, to make stroke care and prevention even better. In 2011 a quality management framework branded Alberta Stroke Improvement was created and funded through Pillar 4. This involved creation of a team of quality improvement consultants who conducted site visits of all primary and comprehensive stroke centres examining stroke services across the continuum.

## Discussion

The APSS demonstrated that with the right effort, it is possible to successfully implement a comprehensive stroke strategy, with improvements in access and quality of care. In Canada the conditions were right for implementation when the APSS began. The Canadian Stroke Strategy had just been created and there was a pre-existing telehealth network although it needed upgrading and expansion to its current condition. Although the APSS benefited from favourable circumstances, much of the progress created was a result of the APSS itself therefore the APSS can serve as a model for other jurisdictions planning improvements in stroke care. Components that contributed to success which could be exported to other strategies are given here.

The APSS had province-wide influence on and control of care processes for stroke across the continuum. This influence was achieved by the comprehensive approach of the strategy. Though this approach professionals across almost all disciplines involved in the care of stroke patients in the province are involved in the program and collaborate. Care points are linked to one another in sequence improving the chances of a smooth care process. Transition points between community and inpatient services, medical team and rehabilitation team, inpatient teams and return to community services in particular have been enhanced. Better use of existing facilities, and the implementation of innovative technologies make the APSS a modern approach to improve care. The ability of the ASC to make final decisions on funding assisted its ability to control and guide the development of stroke care across the province.

Communication between different disciplines, urban and rural centres, the different therapies and the rehabilitation professionals also contributed to the success. Written protocols, the use of evidence-based interventions, such as stroke unit care, and abundant access to clinical stroke specialists are all present in the APSS and are associated with improved outcomes among patients treated for stroke [8]. Telestroke is also an important factor, it reduces travel time and gives experts the ability to cover a larger geographical area. Also important is the ongoing quality management and evaluation plan implemented in the APSS, increasing the chances of continued improvements.

Results on outcomes and quality of care, published in the APSS interim evaluation report [26] are promising. Outcome data were compared to the situation before the introduction of the APSS, and are available because of the thorough data repository of the APSS.

The proportion of patients who arrived within the treatable time window for tPA increased, possibly related to the public awareness campaigns. Knowledge of symptoms decreased in the two years after the campaigns however, highlighting the need for continued public education. The proportion of patients receiving tPA increased due to a more streamlined care process, including the use of additional PSCs, and more patients were discharged back to their pre-admission place of residence. The number of patients referred to a stroke prevention clinic increased, and prescription of preventive medications on discharge increased reflective of better secondary preventive care. Protocols and training of EMS, together with the existence of PSC led to a reduction of the need to transfer stroke patients to comprehensive stroke centres. Patient satisfaction with care remained good.

The government invested 42.5 million Canadian dollars in the APSS over a 5-year period, averaging 8.5 million per year. A detailed economic analysis of the APSS is underway to determine the benefit for cost of the strategy. In the initial three years the actual number of stroke patients admitted to hospital decreased substantially. Whether this reduction can be sustained given the aging demographic of the population is unclear.

Studies and reviews on telestroke projects are difficult to compare, because projects were set up in areas with different needs. Nevertheless, all agree that the number of rural TIA and stroke patients that receive tPA appropriately, increases through telestroke [12, 13]. In Alberta about 23% of telestroke consultations resulted in thrombolytic treatment. In literature percentages range from 1.3% to 30%.

### Strengths and limitations of this study

The availability of many documents, the explanations to them by many professionals and a fourteen days site visit in 2011 form the strength of this study. By using the theories and schemes of Yin we were able to give a systematic view on this innovative case of telestroke services. A limitation of the study is, that we do not offer controllable quantitative data on clinical outcome, utilization of resources, patient’s experiences and costs. In this phase of sciences of telestroke services we can only say for sure: yes, telestroke service works and the results are promising but not yet evidence based.

## Conclusion

In 2005 there was a national initiative to improve stroke care in Canada. In the province of Alberta, this led to the Alberta Provincial Stroke Strategy (APSS). The APSS is a comprehensive approach to improve stroke care across the province. Stroke specialists in tertiary stroke centers initiated the APSS, and worked together with all stakeholders in stroke care in Alberta to make the strategy a success.

To address its goals, the APSS equipped small hospitals for diagnosing and treating acute stroke, introduced telestroke, and enhanced cross disciplinary communication and access to preventive care. Care efficiency is improved by coordinating the care, and integrating uniform use of best practice guidelines, assuring province wide uniformity in good practice. Quality of care improved by better use of stroke prevention clinics and improved multidisciplinary collaboration of health professionals within all disciplines.

Telestroke plays a major role in the approach of the APSS. People living in remote areas now have access to specialist stroke care, for acute care as well as follow up care. Furthermore, education and case conferencing between rural and urban professionals is conducted through telestroke.

The APSS has a clearly defined organizational structure and chain of accountability, with different committees bearing accountability for preventive care, acute care, rehabilitation and evaluation. Based on a blueprint of the strategy the provincial Government gave its operational and financial commitment. The professional content of the strategy is based on national evidence-based guidelines, that were adjusted to the provincial needs in multidisciplinary teams. Data repositories make ongoing outcome and process evaluation possible. An evaluation and quality improvement committee is responsible for ongoing improvements and quality control.

## Reviewers

**Robbert Huijsman**, Professor, Doctor, MBA, Professor Management and Organisation Elderly Care, Institute of Health Policy and Management, Erasmus University Rotterdam, The Netherlands

**Lalit Kalra**, Professor, Department of Neurosciences, Academic Neurosciences Centre, King’s College London

**Ron Heijnen**, MD, Nursing Home Physician and stroke care researcher, Maastricht University, Maastricht, The Netherlands

## Figures and Tables

**Flowchart 1.**
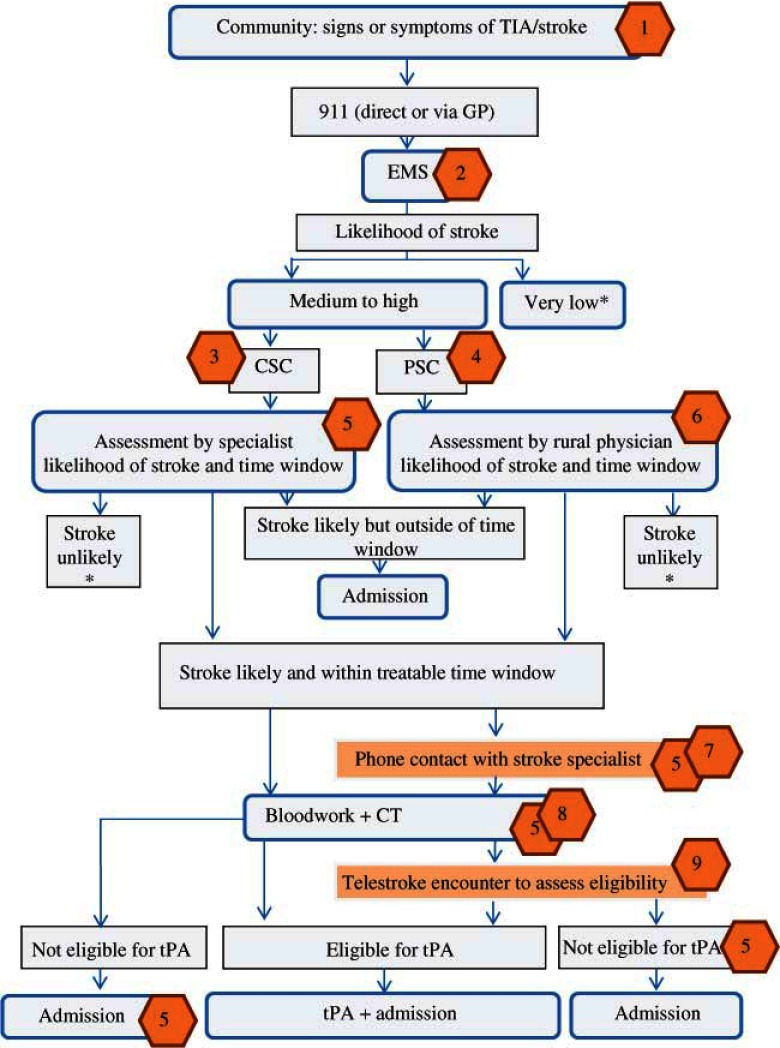
Process flow of a patient with signs of stroke, from community to thrombolysis and admission. *=this process flow is terminated; CSC: comprehensive stroke centre; CT: computed tomography; EMS: emergency medical services; GP: general practitioner; PSC: primary stroke centre; TIA: transient ischemic attack; tPA: tissue plasminogen activator.

**Flowchart 2.**
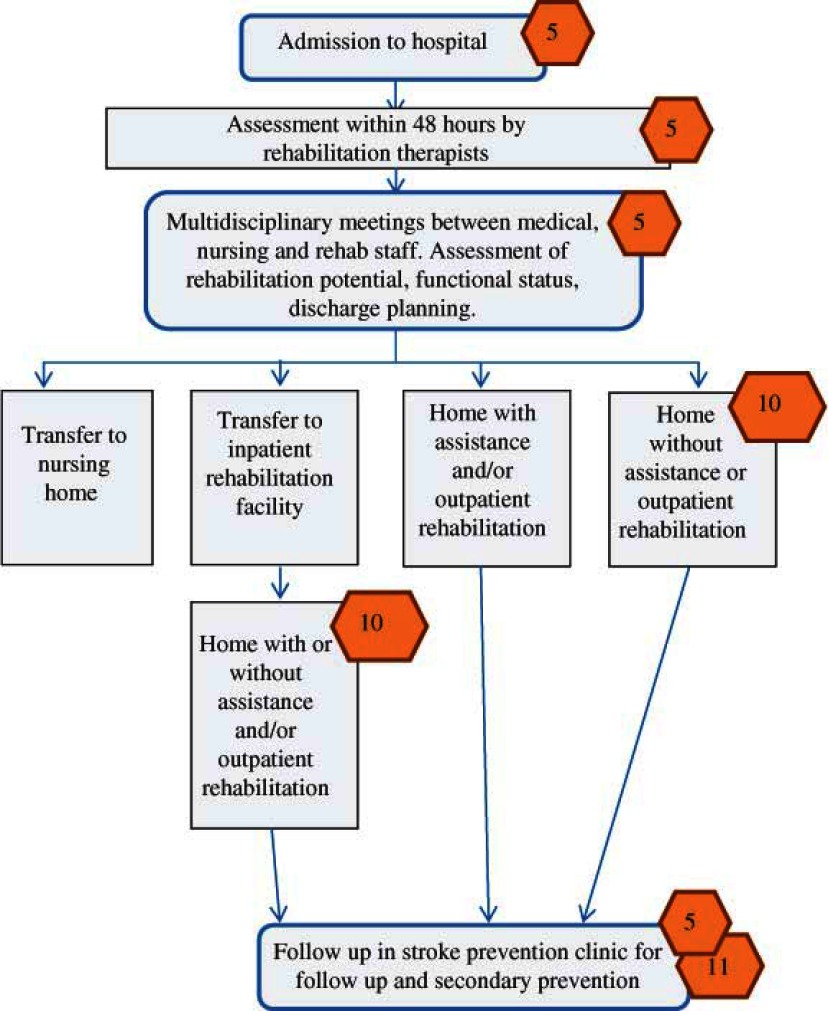
Process flow of a patient with stroke, from admission to community reintegration.

**Table 1.**
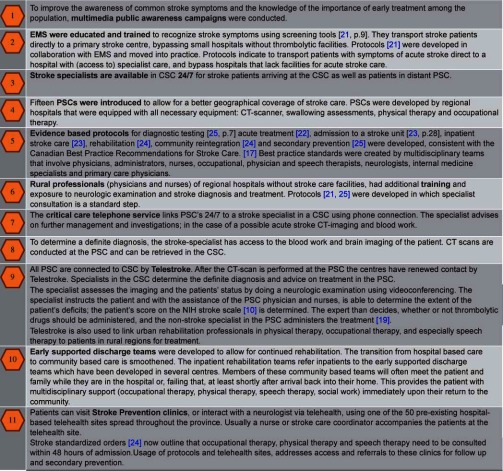
Explanation to flowcharts items the APSS introduced to the stroke service in Alberta, and explanations

**Box 1.**
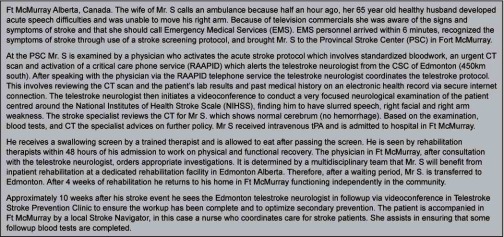
Patient case
